# Authors’ Response to Peer Reviews of “COVID-19 National Football League (NFL) Injury Analysis: Follow-Up Study”

**DOI:** 10.2196/55863

**Published:** 2024-02-13

**Authors:** Troy B Puga, Joshua Schafer, Grace Thiel, Nicholas Scigliano, Tiffany Ruan, Andres Toledo, Prince N Agbedanu, Kevin Treffer

**Affiliations:** 1 College of Osteopathic Medicine, Kansas City University, Kansas City, MO, United States; 2 School of Medicine, University of Kansas, Kansas City, KS, United States; 3 Division of Science, Technology, Engineering, and Math, Department of Health Sciences, Friends University, Wichita, KS, United States; 4 Department of Osteopathic Manipulative Medicine, College of Osteopathic Medicine, Kansas City University, Kansas City, MO, United States

**Keywords:** COVID-19, injury, prevalence, adaptation, sports medicine, follow-up, training, football, epidemiology, sport, athlete, athletic, injuries

*This is the authors’ response to peer-review reports for “COVID-19 National Football League (NFL) Injury Analysis: Follow-Up Study.”*

## Round 1 Review

*Dear authors, the peer review has raised a significant challenge to your statistical analysis by 1 of the reviewers. This feedback will need to be carefully addressed and/or rebutted in order for your paper to proceed at JMIRx Med.*

Response: We believe that our manuscript [1] follows the appropriate format. In addition, we believe that we have addressed the statistical analysis. We also sought outside assistance to help ensure that our biostatistics methods are sound. We also would be willing to accept a transfer to *JMIR Data* if our paper is not accepted in *JMIRx* and a fee waiver can be provided.

In addition, our research team would like to highlight the derogatory, rude, and offensive review by Reviewer AC. While we appreciate the criticisms of our paper and want peer reviewers to remain vigilant in upholding the highest standards, reviewer AC used unprofessional and derogatory language toward our research team with comments such as “their precious paper.” There is no place in academia for derogatory and offensive language, which is part of the guidelines in the “(for reviewers) How to write a high-quality peer review” article on the JMIR website [2].

While we accept the criticisms of our paper, we do not accept the offensive and hostile language that Reviewer AC provided to our research team. We thank you for your concerns and hope this feedback will help guide your peer-review processes in the future.

## Reviewer O [3]

### General Comments

*I would like to commend the authors on performing a follow-up study. This well-executed study provides a good comparison with how COVID-19 disrupted global schedules and the impact it had on the sports sector. The previous version of this study identified how a lack of training greatly increased chances of injuries in National Football League (NFL) athletes. In the current study, the authors have identified how a timely training program can reduce the injury time of athletes. A few points require clarification, such as:*

*1. What is the starting month of the NFL season? The COVID-19 lockdown was implemented from late March 2020 to late May 2020 (mentioned in the current study). Did this fall at the start, at the middle, or toward the end of the training phase of the athletes?*

Response: This fell at the start and through most of the training period. While the lockdown ended in May, most players did not return to in-person activities until the end of July. Preseason games were eliminated and practices in training camp were far fewer in number, with only a 20-day acclimation period [4]. While the official training camp typically only lasts around 2 weeks in the second part of July, minicamps and other unofficial training were eliminated.

The following was added: “While facilities opened back up in May, players were unable to return until the end of July [5]. This shutdown eliminated most of the training period, giving players a narrow 20-day period to reacclimate before the start of the 2020 season [5].”

*2. Were athletes provided any equipment at home, or were they recommended any training protocol by their team’s coaches (the way some clubs in the English Premier League provided gym equipment to their players at home to maintain fitness, or had online practice sessions with their players)?*

Response: Teams were allowed to host virtual instructions and online workouts. They could provide players with up to US $1500 worth of at-home workout equipment [6]. This was optional for players, and they still lacked access to trainers and other department staff and facilities that the NFL is known for having. It can be argued that even at-home workouts are not to the higher standard of facility-based workouts and lack the careful attention and recovery treatments.

The following was added: “It is important to note that although teams could provide players with up to US $1500 worth of at-home training equipment, the players still lacked access to on-site athletic trainers and recovery facilities and were unable to partake in normal preseason training [6]. Any at-home workouts were considered voluntary [6].”

*3. When lockdown restrictions were lifted, how much time were the athletes provided to restore their match fitness?*

Response: About 20 days were provided.

*4. How long does it take for detraining to set in, and how quickly can athletes regain their lost fitness?*

Response: The literature is varied on how long it takes for detraining to set in. Further, detraining can occur at various times when different physiologic changes are considered, such as a change in VO_2max_ and muscle components like mitochondrial proteins. Retraining is similarly widely variable, corresponding to the athlete, pretraining level, time off, and the level of detraining [5]. Due to the wide variability, future research is necessary to determine the exact length of time required for detraining to set in.

We did include discussion of the variability of time to regain lost fitness in the introduction.

#### Minor Comments

*5. Change the tense of some sentences. In the introduction, use “We hypothesized” instead of “we hypothesize” and change the next line to “injury prevalence for the 2021 and 2022 seasons WOULD be lower than the 2020 NFL season...”*

Response: We believe that we have corrected all tense errors.

## Anonymous [7]

*This paper provides epidemiological data on injury incidences in the NFL before and after the COVID-19 lockdown. This paper has the potential to be clinically meaningful; however, it has major flaws that should be addressed before it is reconsidered.*

### Specific Comments

#### Major Comments

*1. The authors state that “This is the first large scale opportunity to demonstrate the effects of these principles and how they are important to understanding injury epidemiology.” However, there are studies that have looked at the effect of COVID-19 on other sporting leagues, for example, Bundesliga, Major League Soccer (MLS), etc. All this has been published and should be cited.*

Response: The Bundesliga study [8] did not include analysis for the years following the pandemic. Another study [9] looked at injury incidence as related to COVID-19 infection. One study [10] looked at a 41-day period of injury epidemiology for 3 periods, 2 of which were after the lockdown but still within the 2020 time period. A study focused on the National Basketball Association (NBA) [11], but again, it only considered injuries for a year following the pandemic lockdowns.

We have changed this sentence in the abstract to address these concerns: “This is the first large-scale and long-term opportunity to demonstrate the effects of these principles and how they are important to understanding injury epidemiology.”

*2. Can the authors please confirm or comment on the potential accuracy of this open data? What validity checks were employed to demonstrate that these data are accurate?*

Response: NFL teams are required to report injuries to players throughout the week [12]. Each team reports injuries weekly, to which individual websites were referenced. The NFL website was used in the event a team report was not available in order to resolve any discrepancies.

The following was added: “Teams are required to report injuries throughout the week of a game, so any and all information should be considered accurate [12].”

*3. How can the authors be sure that the increase in injuries was due to COVID-19?*

Response: We cannot be certain. However, by including a comparison of 2 years prior to the COVID-19 pandemic and 2 years after, we would hope that this would account for any additional changes. We have added a sentence in the limitations to directly address this: “It is also possible that other factors influenced the findings of this study. However, the span of years included would hopefully account for any other possible differences besides the COVID-19–induced lockdown.”

*4. What is meant by injuries? Soft tissue injuries or concussions? Perhaps an analysis on time-loss injuries would be more beneficial and add value*

Response: In our study design methodology, we define injuries as “Contact injuries were included in this study as this is a nonmodifiable risk factor that cannot be controlled due to football being a contact sport [13,14]. COVID-19 infection, sick days, and nonmedical days off were not included in the injury tally. Illnesses were not included in this study, because illnesses are not considered a physical injury and should be reported separately from injuries when performing injury epidemiological studies [13,15].” This includes any soft tissue injuries and concussions. While it might be of interest to assess time-loss injuries, this invites the debate on whether to assess time to return to activity or time to return to full play. Only time to return to activity could be assessed from the publicly available data, and some injuries listed do not fully result in the loss of participation [12]. Further, this was not something considered in the previous study, so we would lack the ability to compare the current findings to the previous study, which was part of the goal of this project.

The following was added: “All other soft tissue injuries and concussions were included during the first week of the associated injury report.”

## Anonymous [16]

*This paper, “COVID-19 NFL Injury Prevalence Analysis, A Follow-Up Study,” is an interesting read. The conclusions drawn in the paper are supported by the data. All the related works are cited appropriately. The limitations of the presented study are discussed appropriately.*

*My only concern is about the 2 histograms. The authors should make these histograms more clear, readable, and engaging. Use standard deviation or error bars wherever applicable.*

Response: Thank you for your comments; we have added error bars to the figures. We have also changed Figure 2 to the outcome measure to make it more appealing.

## Reviewer AC [17]

### General Comments

#### My Review—COVID-19 NFL Injury Prevalence Analysis, A Follow-Up Study

*Throughout the manuscript, including in the title, authors say they analyzed the prevalence of injuries. This is incorrect. They have not analyzed injury prevalence; they did not even collect the data required for such an analysis. Instead, they collected injury incidence data and analyzed them.*

Response: We have removed prevalence from the title and correspondingly changed it to “NFL Injury Analysis.”

*The primary component of this study is analyzing publicly available data to make a conclusion. I have a major concern regarding the statistical analysis the authors have performed. They have collected injury incidence data for each week for each team over the season from publicly available sources. This includes injuries from the same team for each week, which is repeated data. They then calculated the mean per week per team. They had 32 teams and therefore have 32 means for a season. They then compared the mean of those means between seasons using an unpaired* t *test. First, this analysis totally ignores complications due to nonindependence in repeated data. Second, how can we understand the comparison of the means of means? Third, they compared each possible pairs of years. They ignored the multiple comparison issue. This analysis is totally inappropriate. I am not going to accept results of this analysis, or any conclusion based on these results. This is an issue that cannot be rescued by a revision.*

Response: Thank you for your comments. However, a major problem with your review is that at no point were any data repeated, as it is clearly defined in the methods that only new unique injuries were included. There was never any repeat of any data at any point during this study, and therefore, this statement is completely and entirely false. The research team is unaware how this assumption or conclusion was made, as this was clearly stated in the *Methods* section of both the first paper and this paper. Therefore, this claim and the claim of nonindependence is completely false.

The question of the means of means is due to the fact that in 2021, the NFL expanded to an 18-week season, whereas before it was only 17 weeks. While not ideal, the only way to compare to different season lengths is to standardize them by dividing by the number of weeks. This is how we produced this injury rate that we used for comparison. We have added more information into our *Methods* section to ensure that there is no question as to why this was undertaken. In addition, we included the 2018 and 2019 seasons in our new analysis, so you can see that the rate had to be conducted this way due to the fact that 2018, 2019, and 2020 seasons were 17 weeks long, and the 2021 and 2022 seasons were 18 weeks long. All of this reasoning, along with describing how data were not repeated, are clearly laid out in the *Methods* section, and we hope that you will conduct a more thorough reading of this section.

Finally, we have used a Kruskall-Wallis test with Dunn analysis to address the comparison of pairs. The comparisons are statistically significant for 2020 when compared with all the seasons. It is clear that there is significant data here. In addition, comparison of the 2019 season with the 2021 and 2022 seasons did not produce a statistically significant difference, indicating a return to normal levels. We hope that you can accept the results of this analysis. In addition, we consulted with a top leading academic university for biostatistics help in order to ensure that our methods are sound. We hope that our efforts will help you see these results as justified.

*The authors say they have done similar analysis in their precious paper [13]. I now doubt the findings published there too. Unfortunately that paper was also published in* JMIR*. I recommend that editors should consider rereviewing that paper by an independent statistical reviewer.*

Response: Thank you for your comments; however, we would like to highlight that this language is offensive and derogatory and there is no place for comments like this in academia. We appreciate constructive feedback and your dedication to upholding the highest peer reviewing standards; however, we want to make note that this language is offensive and derogatory toward our research team and the previous peer reviewers. The previous paper was reviewed by peer reviewers, who believe that the original analysis was justified.

*There are less severe issues as well. For example, they presented 2 figures—one is redundant in the presence of the other, because the numbers in Figure 1 divided by the number of weeks are the numbers in Figure 2. Further, none of the numbers in any of these figures are the outcome measure they used in the statistical analysis. Therefore, the usefulness of them is limited only to describing the raw data.*

Response: We have retained Figure 1 as it provides the total number of injuries in the data. We have changed Figure 2 to reflect the outcome measure to make it more useful.

*Even if the analysis is correct, they have a fundamental limitation in their interpretation of the results. Their conclusions are based on the underlying assumption that the observed statistical differences were driven by training opportunities. There was no justification for that assumption. How can the authors claim none of the other possible influencing factors changed?*

Response: We cannot confirm or deny that other factors could have influenced these changes, and it is clearly stated in our first paper and this paper within the discussion that other factors could have played a role. However, the largest precipitating change during this time period was COVID-19, which led to limited training opportunities. This is factual evidence and does create a pathway for this epidemiological spike in 2020. It is clearly discussed in both papers within the introductions and discussions that the loss of training can induce detraining, which leads to a predisposition to injuries. This is why there is an epidemiological spike in the number of injuries during 2020 and is statistically significant when compared with the 2018, 2019, 2021, and 2022 seasons. This is also why there is no statistical significance when the 2019 season is compared with the 2021 or 2022 seasons. Therefore, our data and evidence of the events during this time support our conclusions. While other factors could have played a role, the most likely reason was due to these well-documented injuries and strength and conditioning principles.

## Abbreviations

MLS: Major League Soccer
NBA: National Basketball Association
NFL: National Football League
